# Fully Automated Workflow for Integrated Sample Digestion and Evotip Loading Enabling High-Throughput Clinical Proteomics

**DOI:** 10.1016/j.mcpro.2024.100790

**Published:** 2024-05-21

**Authors:** Anders H. Kverneland, Florian Harking, Joel Mario Vej-Nielsen, Magnus Huusfeldt, Dorte B. Bekker-Jensen, Inge Marie Svane, Nicolai Bache, Jesper V. Olsen

**Affiliations:** 1 Faculty of Health Sciences, Novo Nordisk Foundation Center for Protein Research, University of Copenhagen, Copenhagen, Denmark; 2 Department of Oncology, National Center of Cancer Immune Therapy, Copenhagen University Hospital - Herlev and Gentofte, Herlev, Denmark; 3 Evosep Biosystems, Odense, Denmark

**Keywords:** mass spectrometry, workflow automation, plasma proteomics, cancer immune therapy, biomarker discovery

## Abstract

Protein identification and quantification is an important tool for biomarker discovery. With the increased sensitivity and speed of modern mass spectrometers, sample preparation remains a bottleneck for studying large cohorts. To address this issue, we prepared and evaluated a simple and efficient workflow on the Opentrons OT-2 robot that combines sample digestion, cleanup, and loading on Evotips in a fully automated manner, allowing the processing of up to 192 samples in 6 h. Analysis of 192 automated HeLa cell sample preparations consistently identified ∼8000 protein groups and ∼130,000 peptide precursors with an 11.5 min active liquid chromatography gradient with the Evosep One and narrow-window data-independent acquisition (nDIA) with the Orbitrap Astral mass spectrometer providing a throughput of 100 samples per day. Our results demonstrate a highly sensitive workflow yielding both reproducibility and stability at low sample inputs. The workflow is optimized for minimal sample starting amount to reduce the costs for reagents needed for sample preparation, which is critical when analyzing large biological cohorts. Building on the digesting workflow, we incorporated an automated phosphopeptide enrichment step using magnetic titanium-immobilized metal ion affinity chromatography beads. This allows for a fully automated proteome and phosphoproteome sample preparation in a single step with high sensitivity. Using the integrated digestion and Evotip loading workflow, we evaluated the effects of cancer immune therapy on the plasma proteome in metastatic melanoma patients.

## Highlights

• Automated sample prep for LC-MS proteomics for up 192 samples in less than 7 h.
• Integration of digestion and Evotip loading reduces sample input and reagents needed.
• More than 120k quantified peptides in HeLa cells from 1 μg protein on 12 min gradients.
• The workflow can be expanded to include phosphopeptide enrichment.

## Graphical Abstract

**Figure:**
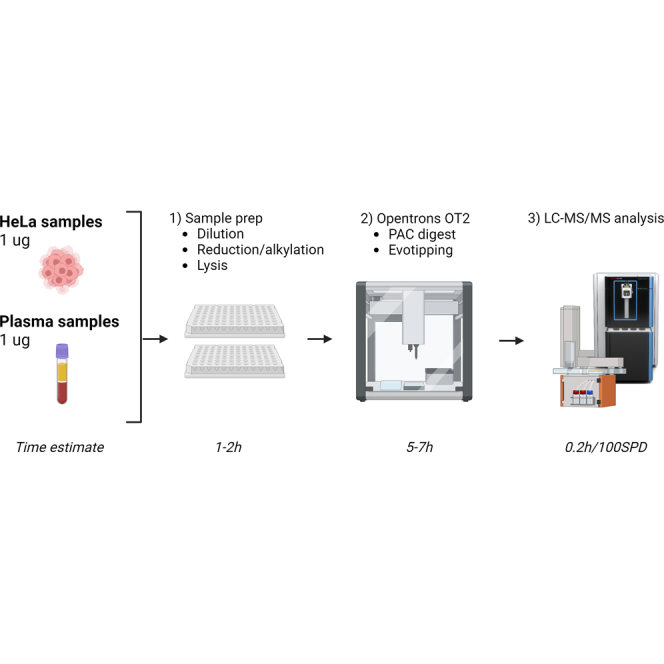

## In Brief

The throughput of shotgun proteomics has been increased significantly and opened up for analysis of large clinical cohorts. Automation of the sample preparation step is highly desirable for robust and cost-effective proteomics and phosphoproteomics analyses. In this manuscript, we show that fully automated proteomics sample preparation is easily achievable with the Opentrons OT2 robot preparing up to 192 samples in parallel with only minimal handling time. We demonstrate the potential of this workflow in a clinical cohort of plasma samples.

In recent years, mass spectrometry (MS)-based proteomics has become a widely used platform for clinical biomarker discovery and studying cellular signaling networks. This has led to technological developments of fast sequencing mass spectrometers that facilitate short online LC-MS/MS enabling high-throughput analyses of hundreds of samples per day (1, 2, 3, 4). It is increasingly feasible to utilize LC-MS/MS for analyzing large patient cohorts but the bottlenecks for large-scale studies have moved to the preanalytical and postanalytical sample processing steps, which are unable to keep up with the increased throughput of the LC-MS/MS analyses.

The preanalytical sample preparation in bottom-up (shotgun) proteomics can crudely be divided into three steps. The first step is preparing the proteins for digestion including protein extraction, cysteine disulfide bond reduction, and alkylation. This step typically involves cell lysis or even tissue degradation and will usually be specific to the sample type in question. The second step is the protein digestion with sequence-specific proteases such as trypsin (5). Sequencing-grade proteases are expensive reagents accounting for most of the cost of proteomics sample preparation, and digestion has traditionally been performed overnight at 37 °C to optimize enzyme efficiency. The third and final step is preparing the resulting peptide mixture for mass spectrometric analysis by acidifying, desalting, and concentrating the sample. The final process has been simplified by sample loading onto an Evotip (Evosep), directly compatible with LC-MS analysis. The EvoTip is a specialized StageTip with reversed-phase C18-based solid-phase material that function as a disposable trap column for desalting and concentrating the peptide samples.

While proteome analysis is the cornerstone of LC-MS proteomics, the increased sensitivity and improvements of methodology has also enabled studying posttranslational modifications (PTMs) such as site-specific phosphorylation or acetylation sites (6, 7). PTMs are mediators of intracellular signaling regulation and by studying these modifications, deep biological insights into cellular dynamics can be attained. Sample preparation for PTM analysis in bottom-up proteomics described above typically requires a PTM-specific enrichment step prior to LC-MS analysis (8, 9).

An obvious way to increase the preanalytical throughput is to automate the sample processing and preparation steps. In addition to increased throughput, automation has the potential to reduce cost and decrease the preanalytical variability, which is essential to minimize for large cohort studies. Semiautomated sample processing workflows for LC-MS/MS–based proteomics have been developed on the Bravo liquid handling platform (Agilent) that works with microchromatographic cartridges that require manual off-board centrifugation steps (10, 11, 12).

Liquid handling robots integrating magnetic bead–based sample preparation, such as the Kingfisher Flex (Thermo Fisher Scientific), enables full automation of the digestion process, but requires relatively large sample input and still involves manual preparation steps before LC-MS analysis (13).

Here, we present a completely automated end-to-end proteomics sample preparation workflow on the Opentrons OT-2 liquid handling robot. A central optimization is the replacement of centrifugation steps with positive pressure that allows the robot to push solvents through the tips. The workflow enables simultaneous preparation of up to 192 samples and encompasses the entire process starting from a cell lysate or protein extract to peptide digests loaded on Evotips ready for LC-MS/MS analysis. The workflow is based on magnetic bead protein aggregation capture (PAC) digestion and can be performed in 6 h and with an option to include magnetic IMAC-based phosphopeptide enrichment (14, 15). Through integration of the digestion process with the Evotip loading, this automation strategy enables protein digestion and loading of almost the entire resulting peptide sample, which greatly increases efficiency while reducing the cost of processing.

## Experimental Procedures

### Sample Preparation

HeLa lysates were prepared from cells cultured in Dulbecco's modified Eagle's medium media, harvested, washed and resuspended in boiling 5% SDS buffer supplemented with 5 mM tris(2-carboxyethyl)phosphine (TCEP) and 10 mM 2-chloroacetamide (CAA) and incubated for 10 min at 99 °C. Protein concentration was measured using a bicinchoninic acid (BCA) assay.

Plasma samples for optimization were collected from anonymous healthy individuals. The blood was drawn into sodium citrate 3.2% tubes (Vacuette, cat# 455322, Greiner BioOne) using a butterfly (Blood Collection Set + Holder 21G × 3/4″, Greiner One Bio, cat# 450085) and immediately centrifuged after collection at 2000*g* for 10 min, followed by another centrifugation of the supernatant at 3000*g* for 10 min to achieve platelet-poor plasma and stored at −80 °C until analysis.

Patients were recruited and the plasma samples were collected at the Department of Oncology at Herlev Hospital. All enrolled patients provided oral and written informed consent before inclusion into the study and was carried out in accordance with the Declaration of Helsinki principles. The study was registered and approved by the local Ethics Committee (H-15007985) at the Capital Region of Denmark. Blood samples were collected at baseline before therapy and after the first series of therapy approximately 21 days after. The blood samples were collected in 9 ml K2-EDTA tubes (Vacuette, cat# 455045, Greiner BioOne), and the plasma was collected after centrifugation at 1300*g* for 10 min within 2 h of sample collection (Platelet-rich plasma) and stored at −80 °C until analysis.

The protein concentration in plasma was approximated to 60 μg/μl. After thawing, the plasma samples were diluted to 225× with PBS and then lysed, reduced, and alkylated with 15 min incubation at 37 °C in 1% SDS, 5 mM TCEP, 10 mM CAA for a final dilution of 300×.

HeLa cell lines for the phosphoproteomics implementation were cultured in Dulbecco's modified Eagle's medium media and seeded into either 96-well or 48-well plates at amounts of 10, 20, 40, or 60 thousand cells per well. Cells were harvested by first washing each well twice in PBS without magnesium and calcium. Boiling 1% SDS buffer supplemented with 10 mM TCEP and 20 mM CAA as well as 5 mM β-glycerophosphate, 5 mM sodium fluoride, and 1 mM sodium orthovanadate was added and the lysate was incubated at 80 °C for 10 min. Lysates were kept in cell culture plates and frozen at −80 °C until further processing. Lysates were thawed at 60 °C and then treated with 20 U Benzonase per well for 10 min at 37 °C. Plates were spun down and lysates were transferred into 96-well PCR plates for further processing in the OT-2.

For drug treatment, 40,000 cells were seeded in 48-well plates and grown for 48 h before treatment with 1 μM anisomycin (CAS No. 22862-76-6, A9789-5MG, Sigma-Aldrich) for 0, 0.5, 1, and 2 h.

### OT-2 Preparation for Sample Digestion and Evotip Loading

The automated PAC workflow is prepared by mixing sample, magnetic beads, and acetonitrile (ACN) in wells of a 96-well sample plate (Eppendorf twin.tec PCR Plate 96 LoBind, cat# 0030129512) (14). The samples were prediluted until the desired protein input could be contained in 5 μl and then transferred to each well in the sample plate. Immediately before starting the protocol on the OT-2, 5 μl magnetic beads (MagReSyn hydroxyl beads, cat# MR-HYX2L, ReSyn Biosciences, Ltd) and 40 μl MS-grade ACN was added to the well for a final aggregation volume of 50 μl.

The samples were processed on an Opentrons OT-2 robot (Opentrons Labworks Inc) using a GEN2 Magnetic Module (#99-00098, Opentrons Labworks Inc). The OT-2 deck positions including the reservoir plate was set up according to the step-by-step guide available at https://www.evosep.com/support/automation-opentrons-ot2 with an enzyme:protein mass ratio of 1:25 for trypsin (Cat# T6567, Sigma-Aldrich) and 1:100 for endoproteinase Lys-C (Cat# 129-02541, FUJIFILM Wako Pure Chemical Corporation) prepared in 50 mM triethyl ammonium bicarbonate. Digestion was carried out at room temperature (RT) using 2 to 4 h incubation time.

Run protocols directly compatible with the Opentrons app (version 7.1.1) was downloaded from the Evosep website (version 1.0) where they are available in an easy-to-use HTML format: https://www.evosep.com/support/automation-opentrons-ot2.

For the phospho-enrichment protocol, 10 μl Ti-IMAC beads (MagReSyn Ti-IMAC HP, cat# MR-TIM010, ReSyn Biosciences) were preprepared in 100% ACN and placed on the right half of the 96-well plate and 30 μl of lysate mixed with 65 μl ACN and 5 μl hydroxyl beads (MagReSyn) was placed in the left half of the plate. A solvent reservoir plate was prepared containing ACN (100% v/v), ethanol (100% v/v), loading buffer (80% ACN, 5% triflouroacetid acid (TFA), 1 M glycolic acid, aq.), wash buffer 2 (70% ACN, 1% TFA, aq.), wash buffer 3 (20% ACN, 0.1% TFA, aq.), elution buffer (1% ammonium, aq.), digestion buffer (100 mM triethyl ammonium bicarbonate), isopropanol (100% v/v), enzyme stocks (trypsin at 1:40, and LysC at 1:80 protease to protein ratio and stored in acidic conditions before dilution for the digest) and Evotip loading buffer (0.1% formic acid aq.). A peptide plate was prepared containing 5 μl of (10% v/v) TFA. For running the phospho protocol, a python script was prepared in jupyter notebook (version 6.48).

After protocol run completion, the peptides, loaded by the robot onto Evotips(EV2011 Evotip Pure, Evosep) were manually moved to an Evosep One for LC-MS/MS analysis.

### LC-MS/MS Analysis

The samples were eluted online using an Evosep One system (Evosep Biosystems) and separated using an Evosep 8 cm (EV1109, Evosep) performance column connected to a steel emitter (EV1086, Evosep) and heated to 40 °C. Gradient methods at 100 samples per day (100 SPD; 11.5 min) 60 SPD (21 min) methods were used.

The HeLa cell samples testing the integrated proteome workflow were analyzed on an Orbitrap Astral Mass Spectrometer (Thermo Fischer Scientific) applying 1800 V spray voltage, funnel radio frequency level at 40, and a heated capillary temperature set to 275 °C. The mass spectrometer was operated in positive mode. Full scan spectra precursor spectra (380–980 Da) were recorded in profile mode using a resolution of 240,000 at *m/z*, a normalized automatic gain control (AGC) target of 500%, and a maximum injection time of 3 ms. The fragment spectra were acquired in data-independent acquisition (DIA) mode, with a precurser mass range of 380 to 980 Da with 2 *m/z* isolation windows without overlap, 299 scan events with a resolution of 24,000. Isolated precursors were fragmented in the HCD cell using 25% normalized collision energy, a normalized AGC target of 500%, and a maximum injection time of 2.5 ms.

The plasma samples were analyzed on an Orbitrap Exploris Mass Spectrometer (Thermo Fisher Scientific) applying 2 kV spray voltage, funnel radio frequency level at 40, and a heated capillary temperature set to 275 °C. The mass spectrometer was operated in positive mode using DIA using a 100 SPD (11.5 min) gradient. The instrument was operated in DIA mode and full scan spectra precursor spectra (350–1400 Da) were acquired with a resolution of 120,000 at *m/z* 200, a normalized AGC target of 300%, with a maximum injection time of 45 ms. Fragment spectra were recorded in profile mode fragmenting 49 consecutive 13 Da windows (1 m/z overlap) covering the mass range 361 to 1033 Da with a resolution of 15,000. Isolated precursors were fragmented in the HCD cell using 27% normalized collision energy, a normalized AGC target of 1000%, and a maximum injection time of 22 ms.

For phosphoproteomics acquisition, the samples were analyzed on an Orbitrap Exploris using a 60SPD (21 min) gradient on a 15 cm EV1109 column with a steel emitter. Full scan spectra precursor spectra (472–1143 Da) were acquired with a resolution of 120,000 at *m/z* 200, a normalized AGC target of 300%, and a maximum injection time of 45 ms. Fragment spectra were recorded in profile mode fragmenting 17 consecutive 39.5 Da windows (1 m/z overlap) covering the mass range 472 to 1143 Da with a resolution of 45,000. Isolated precursors were fragmented in the higher-energy collisional dissociation (HCD) cell using 27% normalized collision energy, a normalized AGC target of 1000%, and a maximum injection time of 86 ms to maximize signal intensity of low intensity phosphopeptides.

### Data Analysis

The MS RAW-files were analyzed using Spectronaut v18.6 (Biognosys AG, Schlieren) with directDIA+ using default search settings against a FASTA file containing the human proteome (SwissProt, 20,402 sequences, with signal peptides removed, downloaded October 2018) and the sequences of the two proteolytic enzymes. For the patient samples, an additional FASTA file containing the protein sequences for the checkpoint inhibitor drugs was included. Carbamidomethyl was set as a fixed modification and N-terminal acetylation and oxidation of methionine as variable modifications. Trypsin was set as cleavage enzyme with a maximum of two missed cleavages. The mass tolerance was set to dynamic in both MS1 and MS2 level with a correction factor of 1. A Q value of 1% against mutated decoys was applied to filter identifications with a false-discovery rate level of 0.01 at both the peptide and protein level. Quantification was performed using the automatic setting and normalized with the integrated cross-run normalization feature unless otherwise specified. The method evaluation feature was applied when comparing different sample preparations within the same search. Phosphoproteomics data was additionally searched using phosphorylation (STY) as a variable modification in the PTM tab, with a localization probability cut-off of 0.75.

The data analysis was performed in R version 4.2.2 (R Core Team (2021), R: A language and environment for statistical computing, R foundation for statistical computing) with R Studio 2023.09.1 Build 494. The patient data was normalized using the variance stabilization normalization using vsn package (16). For statistical purposes, missing data in the patient samples were imputed using the MissForest package after filtering out proteins found in less than 30% of all samples (17). The outcome groups were compared by comparing the log2 of the ratio after/before therapy using a linear model from the limma package (18). *p* values were adjusted using the Benjamini–Hochberg procedure. The quantified plasma proteins were annotated using a manual adaption of the human secretome found at the human protein atlas (19).

Phosphorylation site stoichiometry information was calculated using a plugin implemented into the Perseus platform (rapid and site-specific deep phosphoproteome profiling by DIA without the need for spectral libraries) (20). The exported data was processed using the dapar/Prostar package (21). First, the data was filtered for missingness below 25% in all samples. Data was normalized using the variance stabilization normalization. Missing values were imputed using the slsa algorithm for partially observed values and determined quantile imputation was done for data points missing in entire conditions (22). Regulated sites were identified using student’s *t* test between each condition with log2 fold change cut-off at 0.5. *p* values were adjusted using the Benjamini–Hochberg algorithm. Kinase activity estimation was done for regulated sites using the ROKAI tool (23).

The MS proteomics data have been deposited to the ProteomeXchange Consortium *via* the PRIDE partner repository with the dataset identifier PXD048325 (24).

### Experimental Design and Statistical Rationale

The aim of the study was to assess performance and reproducibility of the robotic workflow. To this aim, the variability between identical samples run in parallel with the same protein digestion and Evotip loading workflow (termed workflow replicates) were compared. The number of replicates was primarily determined by practical considerations including number of conditions, plate size, and available instrument time. Statistical tests were not used to assess differences in performance between different workflow replicates.

The number of biological replicates for the application test was limited by availability of appropriate samples in the biobank. From the available samples the first 96 (48 patients) were chosen to fit the 96-plate format which allowed detecting effect sizes down to 0.3 on log2 scale with a power of 80% when assuming a median SD of 0.5 in the baseline group.

## Results

### Sample Preparation on the Opentrons OT-2 Robot

**Fig. 1:**
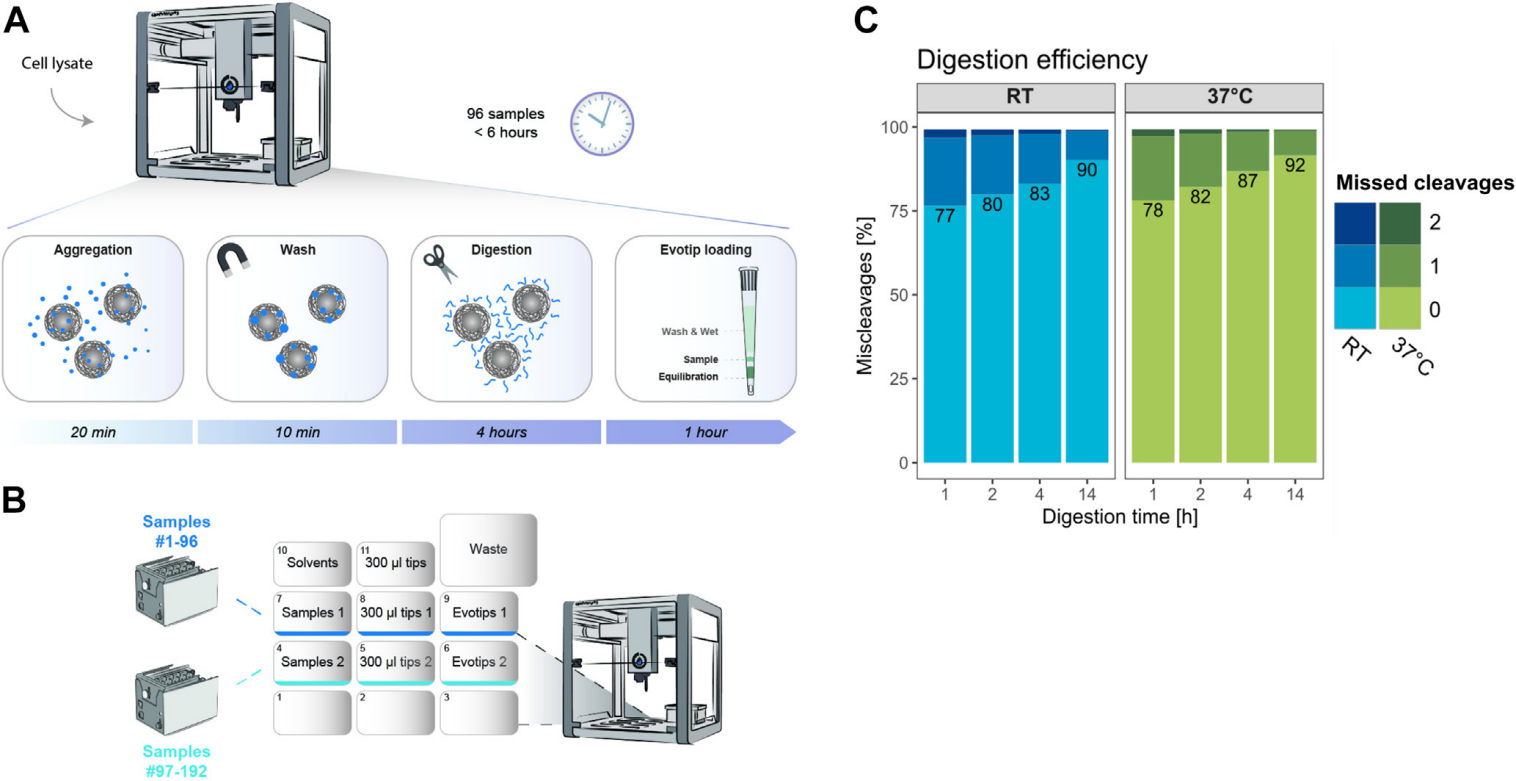
**Overview of the automated workflow.***A*, schematic overview of the integrated workflow on Opentons OT-2 robot. *B*, deck layout for both 96 and 192 samples of the OT-2 before digestion. *C*, comparison of digestion efficiency at different temperatures and incubation periods.

The fully automated proteomics sample preparation workflow was implemented on the OT-2 to process up to 192 samples in parallel within 6 h. This involved 20 min PAC on magnetic beads, 10 min buffer exchange and washing, 2 to 4 h on-bead protease digestion at RT, and finally 60 min Evotip loading (Fig. 1*A*). The OT-2 robot was chosen as it is flexible and accessible to most laboratories due to its low cost and straightforward programming control interface. The layout of the OT-2 deck for automated sample preparation of proteome samples includes buffers, Evotips, and limited usage of pipetting tips to avoid manual intervention (Fig. 1*B*). Traditionally, tryptic digestion prior to LC-MS/MS analysis has been carried out at 37 °C and overnight to ensure complete digestion, which in the current setup would require either a heating module or implementing a manual step to move the sample plate to a heating device (25). Instead, to minimize manual intervention and maximize throughput, we tested the digestion efficiency on HeLa cell line lysates when combining endoproteinase Lys-C and trypsin at different temperatures and with much shorter digestion periods. We found that the overnight digestion performed equally well at RT as at 37 °C achieving ∼10% missed cleavage sites (Fig. 1*C*). At shorter time periods, digestion at 37 °C was marginally better than RT with the most pronounced effect at the shortest time period of 1 h. Thus, our experiment demonstrated that short 2 to 4 h digestion periods at RT is feasible and only a minor compromise in digestion efficiency which is in line with what others have reported (26).

### Performance in HeLa Cell Lines

We evaluated the automated workflow using two full plates of HeLa cell lysate, which is a well-known cell type and frequently used standard model in LC-MS/MS-based proteomics for quality control. For matching the high-throughput of the automated workflow with LC-MS/MS analysis measurement speed, we tested the performance using a 100 SPD method (11.5 min active LC gradient) on the Orbitrap Astral mass spectrometer operated in narrow-window DIA mode (27). The script loaded on the Opentrons app is provided in the Supplementary Material.

**Fig. 2:**
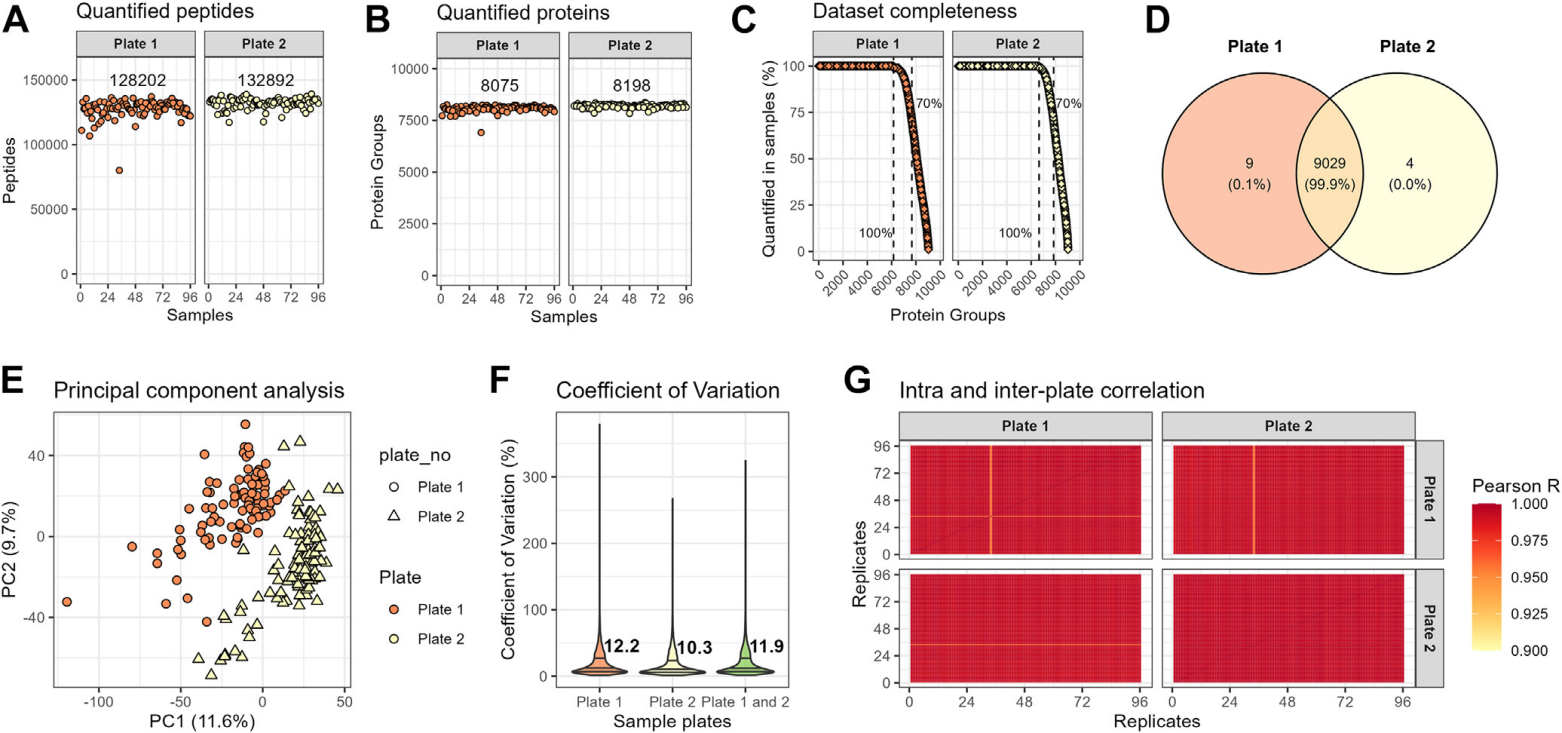
**Performance across 192 HeLa samples.***A*, count of identified peptides across two plates (192 samples) of HeLa lysate at 100 SPD (11.5 min) gradient on the Orbitrap Astral. *B*, count of unique protein groups across two plates (192 samples) of HeLa lysate at 100 SPD (11.5 min) gradient on the Orbitrap Astral. *C*, dataset completeness across 192 samples and 9047 protein groups. *D*, *Venn diagram* of protein groups identified on plate 1 and plate 2. *E*, principal component analysis of the 192 HeLa samples across two different sample plates. *F*, *violin plot* of the CV within and across the two plates with the median CV provided. *G*, Pearson correlation coefficients between the 192 samples and two sample plates. SPD, samples per day.

**Supplemental Figure S1:**
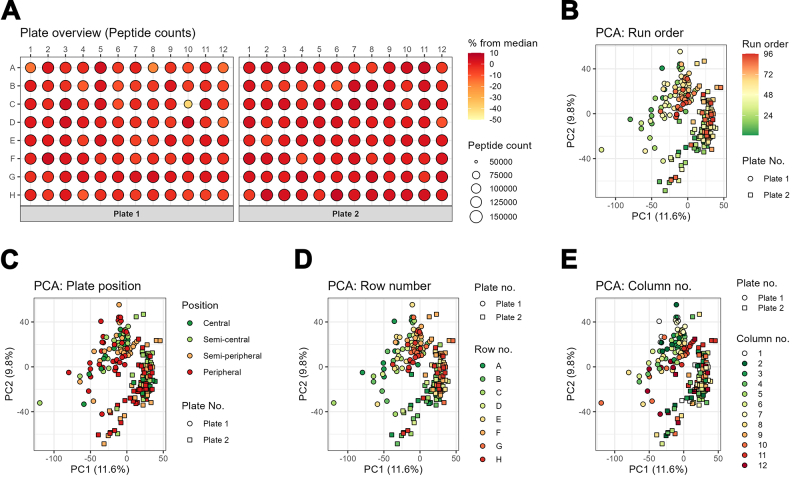
**Inter-plate reproducibility.** Evaluation of 192 identical HeLa samples loaded on two plates with 1 μg HeLa and analysed at a 100 SPD (11.5 min) gradient on an Orbitrap Astral. *A*, peptide counts across plate locations. *B*, principal component analysis of run order on the mass spectrometer. *C*, principal component analysis of the central-peripheral sample position in plate. *D*, principal component analysis of the row number in plate. *E*, principal component analysis of the column number in plate.

The HeLa cell lysate protein concentration was measured to 4 μg/μl on a BCA assay, and we prepared 5 μl of a 20× dilution equivalent to 1 μg total protein input per sample with a 50% load onto the Evotip. In these samples, we were able to quantify approximately 130,000 peptides and 8000 proteins across the 192 samples (Fig. 2, *A* and *B* and supplemental Fig. S1*A*). A list of the quantified proteins and the median quantity can be found in the supplemental Table S1. The two plates were almost identical in terms of dataset completeness, protein group identifications (Fig. 2, *C* and *D*). The plate number could be distinguished in a principal component analysis (Fig. 2*E*). The protein quantifications within and between the two plates were very comparable in terms of coefficients of variation and were highly correlated based on the high Pearson correlations indicating low intraplate and interplate variability (Fig. 2, *F* and *G*). In a single sample, located in position C10 on plate 1, the peptide and protein quantifications were about 15 to 20% lower than the median (supplemental Fig. S1*A*). We evaluated but did not find systemic effects toward run order, border-proximity, row, and column number when assessed by principal component analysis (supplemental Fig. S1, *A*–*E*).

### Storage Stability on Evotips

**Fig. 3:**
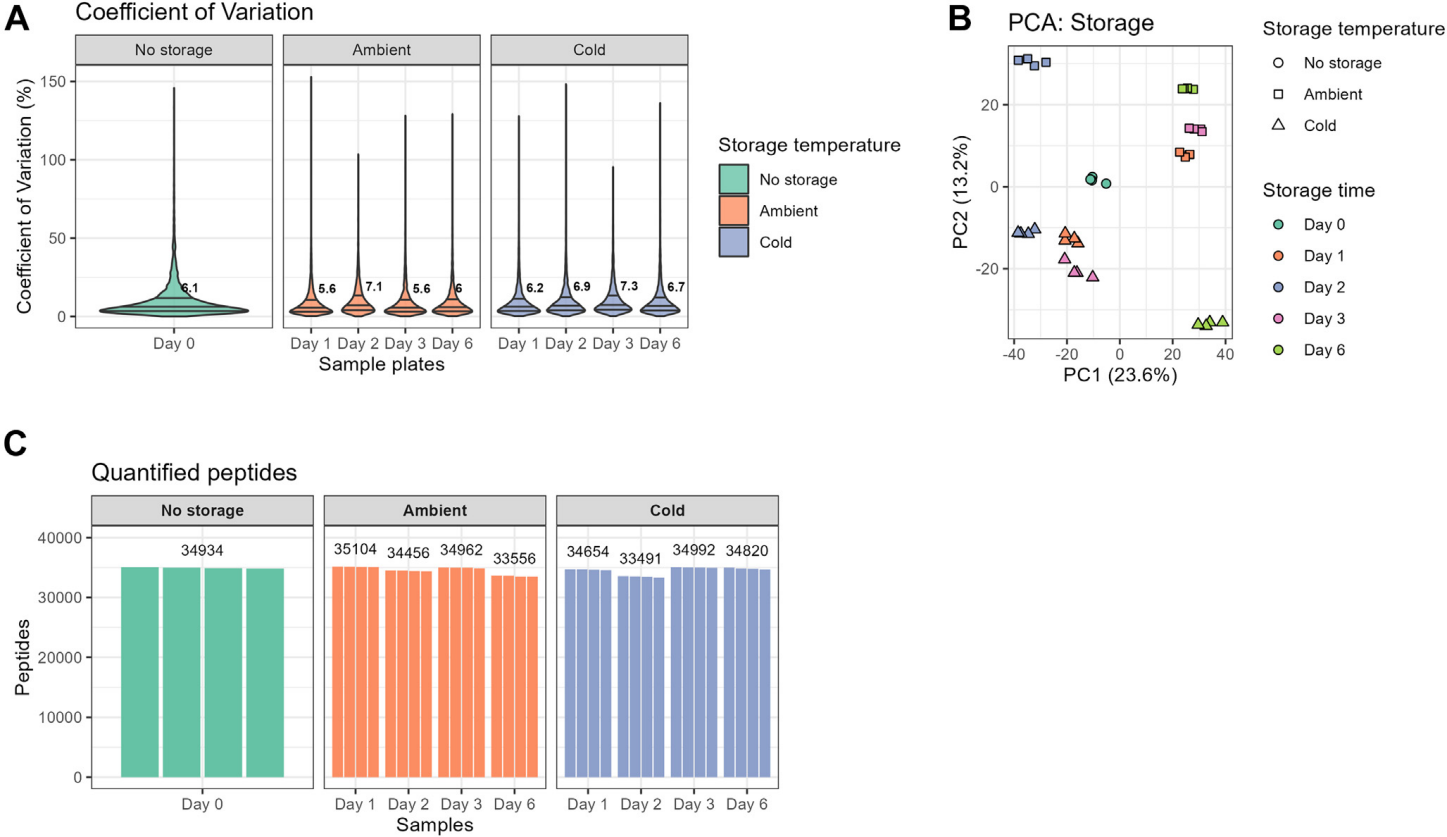
**Storage stability after the automated workflow.***A*, count of identified peptides at day 0 and after 1 to 6 days of storage at room temperature on the instrument (*ambient*) or refrigerated until analysis (*cold*) analyzed at 100 SPD (11.5 min) gradient on a Orbitrap Exploris. *B*, CV of identified protein groups before and after storage. *C*, principal component analysis comparing no storage to ambient and cold storage after 1 to 6 days. SPD, samples per day.

Samples ready for LC-MS/MS cannot always be analyzed immediately after sample preparation and a convenient workflow would allow sample storage until MS measurement becomes available. Since the final step in the automated workflow is sample loading on Evotips, the possibility to store a peptide digest is limited by its stability on the Evotip. We tested the impact of storing samples either cold (4 °C) or at ambient temperature (23 °C) up to 6 days before the LC-MS injection. The number of quantified peptides and their CVs remained stable through the testing period at both storage conditions (Fig. 3, *A* and *B*). In a principal component analysis, the samples also clustered together according to storage temperature and storage duration. We observed that the refrigerated samples clustered close to the unstored samples after 1 to 3 days but moved in the direction of the ambient stored samples at day 6. The samples stored at ambient temperature clustered together through the storage period with the exception of day 2 (Fig. 3*C*).

### Performance in Plasma Samples

**Fig. 4:**
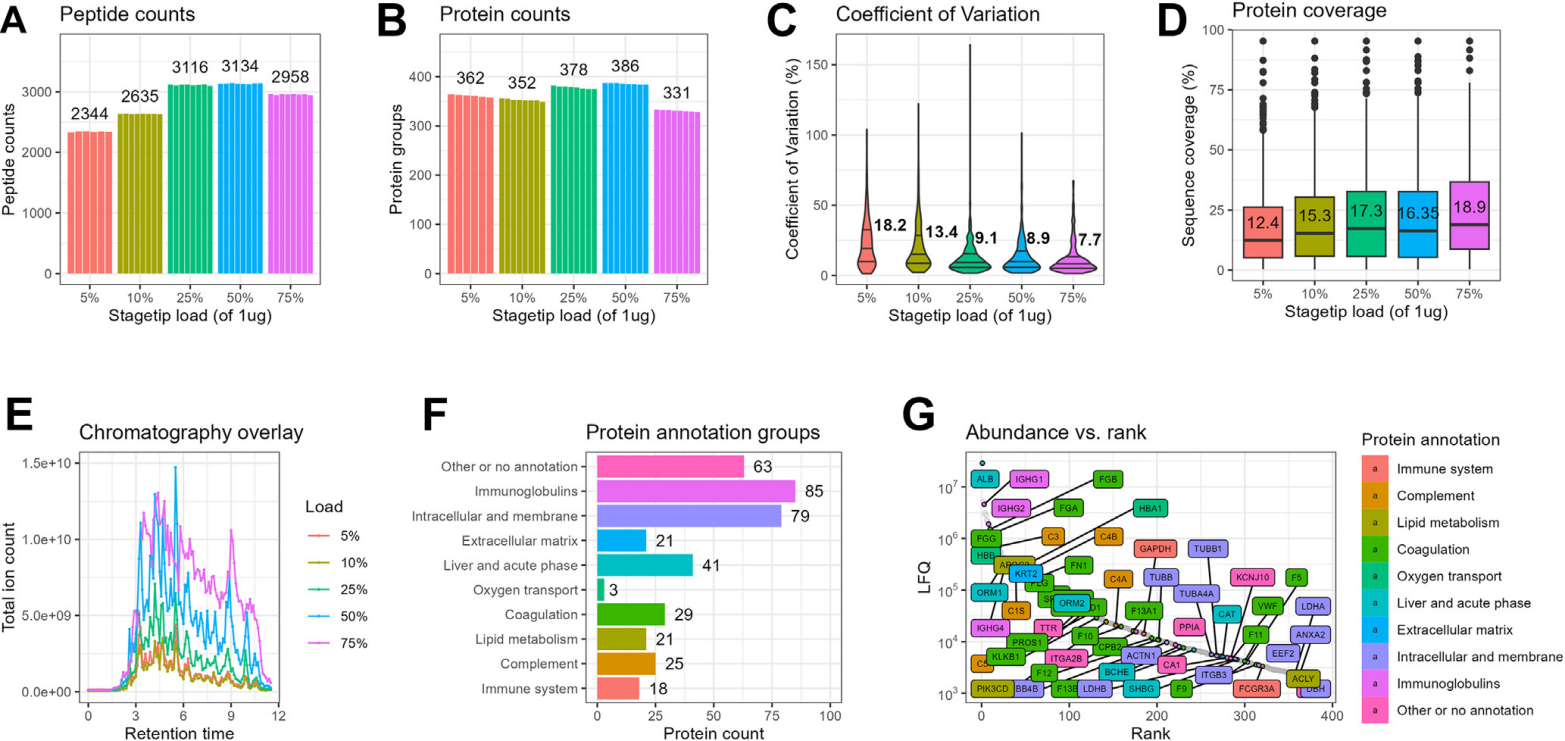
**Performance in plasma samples.***A*, identified peptides with 1 μg plasma input using different Evotip loading masses analyzed at 100 SPD (11.5 min) gradient on a Orbitrap Exploris. *B*, identified protein groups with 1 μg plasma input using different Evotip loading masses. *C*, *violin plot* of the CV across different loading masses with the median CV provided. *D*, relative protein sequence coverage with different loading masses. *E*, overlay of the total ion intensity (TIC) *versus* retention time across different Evotip loads. *F*, annotation overview of identified plasma proteins (25% Evotip load) adapted from the proteinatlas.org. *G*, relative abundance rank *versus* median label-free intensity (LFQ) of identified protein (25% Evotip load). The Food and Drug Administration-approved drug targets are labeled and colored according to their protein annotation. SPD, samples per day.

Automated sample preparation is a prerequisite for handling and analyzing large-scale clinical cohort studies. Clinical proteomics of large patient cohorts often involves analysis of blood plasma. Plasma is a rich source of proteins and represents systemic physiology. The extreme dynamic range of plasma proteome of ∼12 orders of magnitude is a key challenge for LC-MS/MS based analyses, but plasma remains an attractive source of biomarkers that is routinely collected in most clinical cohorts. We tested the performance of the automated workflow with plasma samples using 1 μg of protein input with the 100 SPD (11.5 min) gradient on the Orbitrap Exploris. The maximum number of identified peptides and proteins were 3134 and 386, respectively (Fig. 4, *A* and *B*), which is comparable to state-of-the-art DIA analysis in undepleted plasma using short LC gradients (1, 28, 29). The best overall performance was seen with loading 25% and 50% of the sample input (∼250 ng and ∼500 ng). The loss in performance at high loads is possibly caused by column saturation with highly abundant peptides. This trend was also confirmed when comparing the CV and protein sequence coverage of the different sample loads where 250 ng was slightly better than 500 ng (Fig. 4, *C* and *D*). The intensity of the MS signal correlated to the sample load confirming the actual difference in sample load (Fig. 4*E*). We are able quantify proteins within multiple different functions many of which were listed as drug targets by the United States Food and Drug Administration (Fig. 4, *F* and *G*). A list of the quantified proteins, their annotation as well as their median quantity can be found in supplemental Table S2.

### Clinical Application in Cancer Immune Therapy

**Fig. 5:**
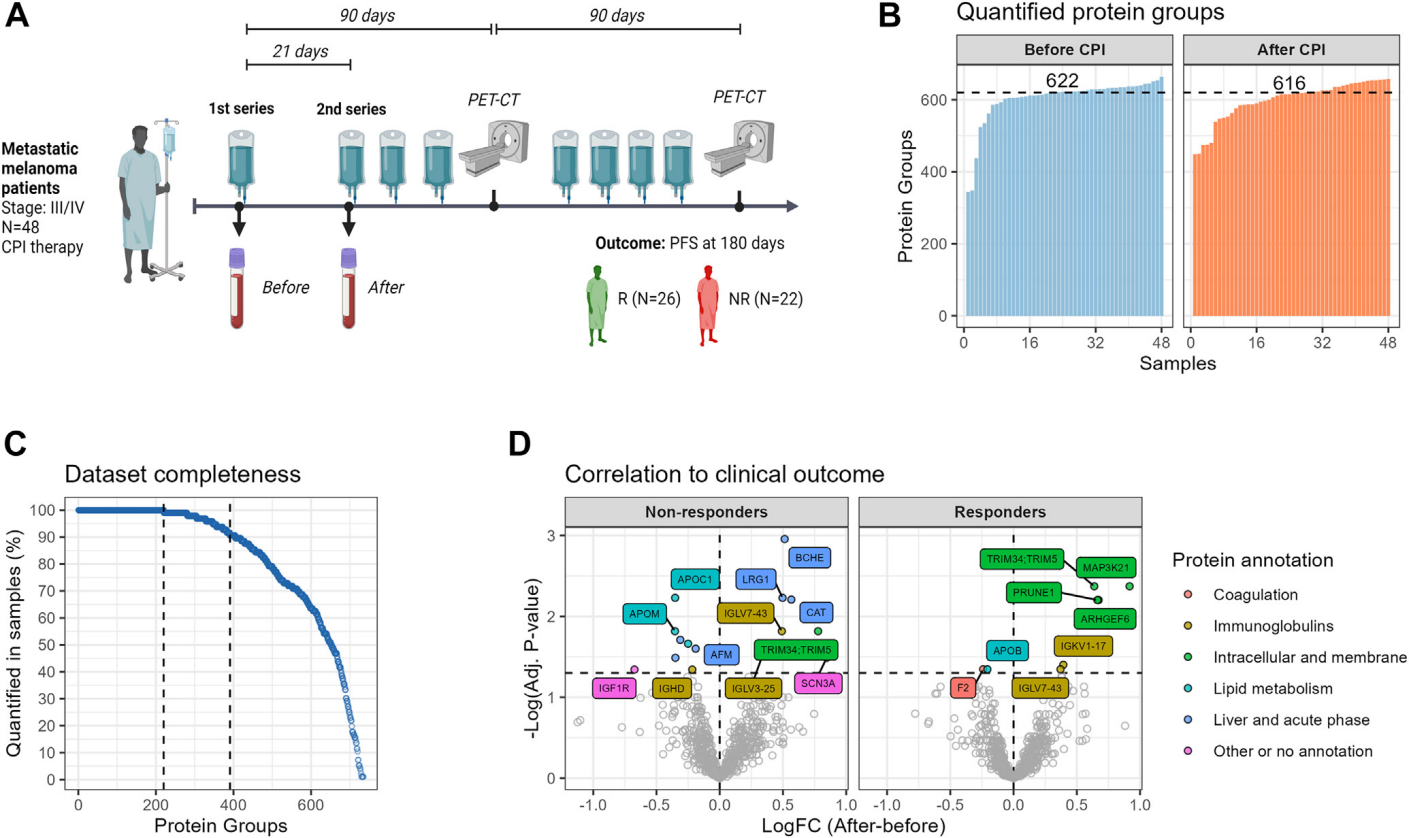
**Application in clinical cohort of metastatic melanoma patients.***A*, overview of study design, patient inclusion, and evaluation. The melanoma patients were recruited prior to checkpoint inhibitor therapy where the first sample was collected (before CPI) and a second blood sample was collected approximately 21 days after the first therapy (after CPI). Clinical response to therapy was determined as progression-free survival of more than 180 days encompassing two routine evaluation scans. *B*, count of quantified proteins across all patients at both time points. *C*, dataset completeness assessment visualizing the protein groups identifications across the dataset. *D*, *volcano plot* comparing the time points (after CPI−before CPI) within responding and nonresponding patients. The significant proteins are colored and labeled according to their protein annotation.

To test the automated workflow in a clinical setting, we analyzed the plasma proteome from a patient cohort consisting of 48 metastatic malignant melanoma cancer patients with paired plasma samples collected in a biomarker study (Table 1 and Fig. 5*A*). The patient samples were collected before and 3 weeks after initiating of immune therapy with checkpoint inhibitors as standard therapy. The outcome of the therapy was assessed with PET-computed tomography after 3 and 6 months.

**Table 1:** Patient characteristics

Characteristics	Total (N = 48)	Response (PFS >180 days)	
		Nonresponders (N = 21)	Responders (N = 27)
Age (years)			
Mean (CV%)	68.5 (20.9%)	66.1 (26.9%)	70.3 (15.5%)
Median [Min, Max]	72.5 [27.0, 86.0]	73.0 [27.0, 86.0]	72.0 [44.0, 86.0]
Sex			
Male	36 (75.0%)	15 (71.4%)	21 (77.8%)
Female	12 (25.0%)	6 (28.6%)	6 (22.2%)
Melanoma type			
Skin	41 (85.4%)	19 (90.5%)	22 (81.5%)
Ocular	1 (2.1%)	1 (4.8%)	0 (0%)
Unknown focus	6 (12.5%)	1 (4.8%)	5 (18.5%)
Melanoma stage			
III	3 (6.3%)	1 (4.8%)	2 (7.4%)
IV M1a	5 (10.4%)	3 (14.3%)	2 (7.4%)
IVM1b	15 (31.3%)	7 (33.3%)	8 (29.6%)
IVM1c	17 (35.4%)	8 (38.1%)	9 (33.3%)
IVM1d	7 (14.6%)	2 (9.5%)	5 (18.5%)
N/A	1 (2.1%)	0 (0%)	1 (3.7%)
BRAF mutation			
WT	30 (62.5%)	12 (57.1%)	18 (66.7%)
Mutated	17 (35.4%)	8 (38.1%)	9 (33.3%)
N/A	1 (2.1%)	1 (4.8%)	0 (0%)
PD-L1 expression			
Negative	27 (56.3%)	14 (66.7%)	13 (48.1%)
Positive (>1%)	20 (41.7%)	6 (28.6%)	14 (51.9%)
N/A	1 (2.1%)	1 (4.8%)	0 (0%)
Cancer therapy			
Anti-PD1	32 (66.7%)	12 (57.1%)	20 (74.1%)
Anti-PD1/CTLA4	14 (29.2%)	7 (33.3%)	7 (25.9%)
Anti-CTLA4	2 (4.2%)	2 (9.5%)	0 (0%)
Best overall response rate			
PD	18 (37.5%)	18 (85.7%)	0 (0%)
SD	11 (22.9%)	3 (14.3%)	8 (29.6%)
PR	13 (27.1%)	0 (0%)	13 (48.1%)
CR	6 (12.5%)	0 (0%)	6 (22.2%)
Progression-free survival (days)			
Mean (CV%)	358 (74.4%)	114 (43.0%)	548 (36.9%)
Median [Min, Max]	287 [22.0, 872]	122 [22.0, 187]	489 [195, 872]
Overall survival (days)			
Mean (CV%)	555 (43.7%)	397 (60.5%)	677 (23.9%)
Median [Min, Max]	597 [69.0, 923]	331 [69.0, 813]	687 [395, 92]

Clinical overview of patients with metastatic malignant melanoma.

Abbreviations: OS, overall survival; PFS, progression-free survival.

Across all neat plasma samples, we were able to quantify a median of 622 protein groups with approximately 200 proteins quantified in all samples and 400 proteins quantified in 90% of the samples (Fig. 5, *B* and *C*). A list of the quantified proteins and the median quantity can be found in the supplemental Table S3.

To evaluate the potential for biomarker discovery in this patient cohort, we compared the dynamics in protein quantifications from before to after initiating of therapy in responding patients *versus* nonresponding patients (Fig. 5*D*). These comparisons showed that nonresponding patients had increases in three liver- and acute phase proteins (BCHE, CAT, LRG1) and decreases in several apolipoproteins (APOA1, APOC1, APOM). High LRG1 levels have previously been reported as a poor prognostic marker in malignant melanoma and associated with disease relapse in the neoadjuvant setting (30). Similarly decreases of several apolipoproteins (APOB, APOE, APOM) in nonresponding patients have also been reported as prognostic markers for malignant melanoma (29). Responding patients also had significant increases in several intracellular proteins (ARHGEF6, PRUNE1, MAP4K21, TRIM34, TRIM5). ARHGEF6 has been linked to T cell migration in lung cancer (31), while MAP3K21 (or MLK4) has also been implicated in immune infiltration in cervical cancer (32, 33). This analysis demonstrates the potential for biomarker discovery with clinical proteomics samples using the fully automated sample preparation workflow.

### Phosphoproteomics

**Fig. 6:**
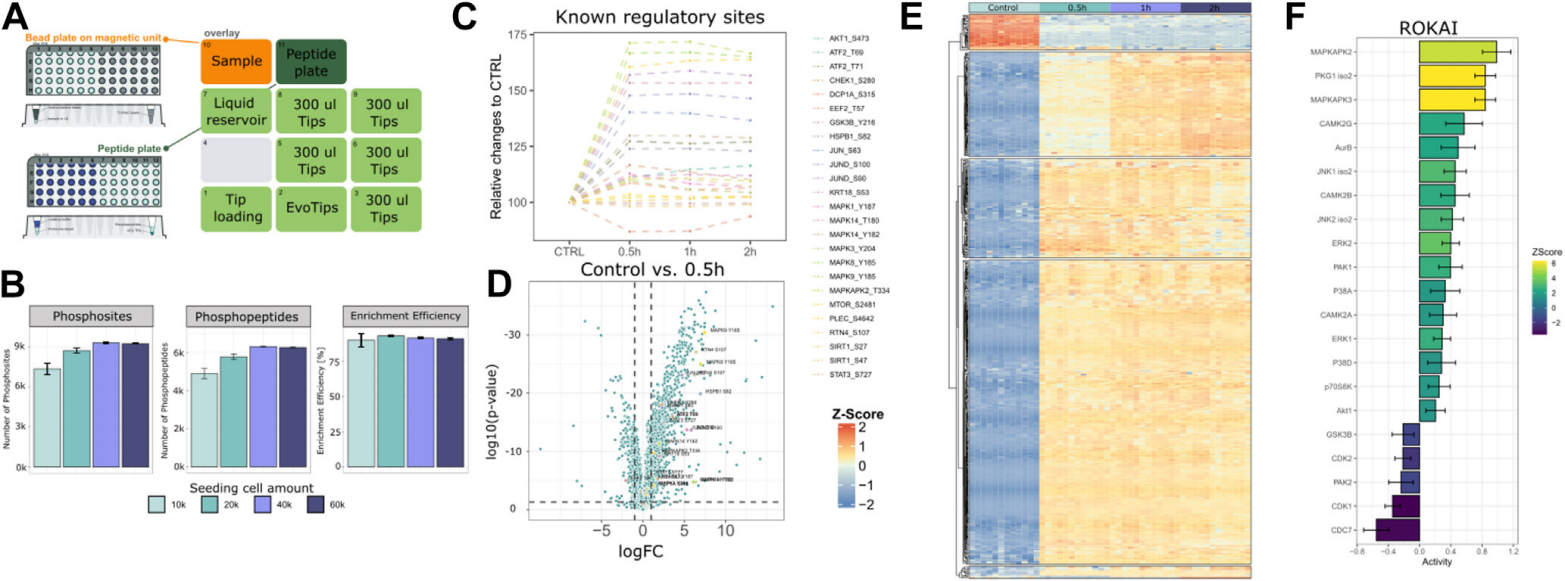
**Application of phosphoproteomics.***A*, overview on the OT-2 setup for phosphoproteomics. *B*, initial benchmarking of the using HeLa cell lines. In total 10,000 and 20,000 cells were seeded in a 96-well format and 40,000 and 60,000 were seeded in a 48-well format and performed in triplicates. *C*, relative changes of known interactors to anisomycin treatment, based on PhosphositePlus annotated sites. *D*, *volcano plot* of the control condition against 0.5 h of treatment. Known interactors are labeled. *E*, heatmap of regulated sites with >2 log FC for the different time points. *F*, Rokai plot of regulated sites for the 0.5 h time point against control. log2FC, log2-fold change.

Global analysis of site-specific protein phosphorylation status provides a different view of the proteome by directly informing on the activity states of signaling pathways and networks (34). However, as phosphorylation is a substoichiometric modification, it typically requires implementation of specific phosphopeptide enrichment strategies in the sample preparation workflow (6). To do this, we modified the configuration of the OT-2 protocol to incorporate an automated phosphopeptide-enrichment step after the peptide extraction and digestion. While a small part of the peptide digest was loaded on Evotips for proteome analysis, the remaining sample was enriched for phosphopeptides and subsequently loaded on Evotips for phosphoproteome analysis. The 96-well plate located in the magnetic unit housing the magnetic beads was used for both the proteome digestion and the phosphopeptide enrichment, allowing 48 samples per plate. A peptide plate was prepared for the processing of peptides in between the digest and the phosphopeptide enrichment and acidification of the phosphopeptides after elution in basic ammonia buffer (Fig. 6*A*).

**Supplemental Figure S2:**
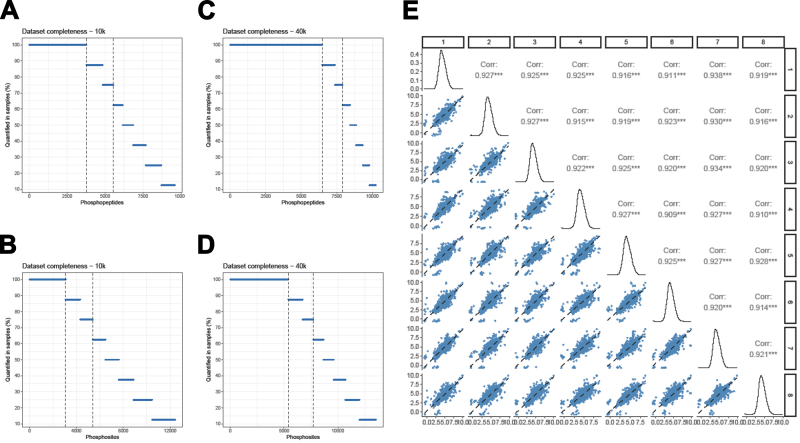
**Data Quality of the phosphoproteome.** Dataset completeness for phospopeptides and phospho-sites across the replicates in 10k seeding (*A* and *B*) and 40k seeding (*C* and *D*). *E*, correlation plot and Pearson coeffiencts between replicates in the 40k seeding.

To test the performance and sensitivity of this approach, we seeded 10,000 or 20,000 HeLa cells per well (in triplicates) in a 96-well plate and 40,000 or 60,000 cells per well in a 48-well plate, respectively. The cells were harvested after 2 days after seeding and subsequently processed with the phosphoproteomics protocol on the OT-2. In all conditions, we were able to identify more than 7000 phosphosites and 4000 phosphopeptides, with 40,000 cells in a 48-well plate giving the best results with >8000 phosphosites reproducibly identified, which are similar to what would be expected from this setup (supplemental Fig. S2) (10, 35). Sample acidification and dilution of the digest directly in the binding buffer was sufficient for phosphopeptide enrichment using Ti-IMAC beads, resulting in enrichment efficiencies of >90%, without any C18 cleanup prior to enrichment (Fig. 6*B*).

To fully assess the potential of an automated phosphoproteomics workflow for high-throughput cell signaling studies, we carried out a large-scale experiment analyzing dynamic phosphorylation sites in response to anisomycin treatment. Anisomycin is a well-described bacterial antibiotic that inhibits protein synthesis and induces a cellular stress response involving protein kinases such as c-Jun N-terminal kinase (JNK) and p38 (36). After drug incubation for up to 2 h, we could see clear temporal phosphorylation dynamics of known anisomycin-regulated sites and many sites were markedly upregulated already within half an hour of stimulation (Fig. 6*C*). To assess reproducibility of the workflow, we compared the 0.5 h anisomycin treatment time point with the unstimulated control across 12 replicate samples (Fig. 6*D*). We found >1000 significantly regulated sites including upregulated sites on known downstream targets of JNK and p38. Unsupervised k-means clustering of all regulated sites showed distinct clusters related to the activation or inhibition of phosphosites over time (Fig. 6*E*). A Rokai analysis pinpointed the changes in predicted kinase activity associated to p38 and JNK downstream signaling as expected by the drug activity (Fig. 6*F*).

## Discussion

Fast and reliable sample preparation is becoming increasingly important for bottom-up proteomics with the continuous improvements of sensitivity and throughput of the LC-MS/MS platform. Poor sample preparation results in inaccurate and irreproducible measurements or introduces contaminants that can interfere with the downstream analysis. Even when stringently performed, manual sample preparation is laborious and prone to interinvestigator heterogeneity. Heterogeneity could be minimized by automation, which would also potentially reduce cost and increase throughput.

Here, we presented a fully automated, hands-off, end-to-end proteomics sample preparation workflow based on the versatile and affordable Opentrons OT-2 platform. The workflow combines the PAC digestion using magnetic microbeads with automated loading of resulting peptides directly on Evotips reproducible desalting and storage. The workflow requires no manual intervention after the initial preparation, resulting in ready-to-analyze samples in approximately 6 h for up to 192 samples in parallel. Overall, our results demonstrated that the workflow is reproducible and stable with highly acceptable level of proteome depth. We found a single outlier in our dataset that appeared random and could also be a manual error during sample preparation of the many replicates.

With immediate the capture and storage of peptides on Evotips, the protocol offers an alternative to bulk material preparation by emphasizing efficient utilization of the sample. This greatly reduces the amount and cost of sequencing grade proteases for digestion while preserving the unprocessed sample for later usage. For short gradient plasma proteome analysis, we and others have measured protein counts of ∼220 at 5 min/180 SPD (1) and 259 at 12 min gradients/100 SPD (25). In these studies, undiluted plasma was digested and the amount of trypsin used per sample was 1.2 μg and 3 μg, which are up to 15 to 30× more than what is needed with our protocol (0.05 μg/sample or 8 μg/plate). Furthermore, we see that the loading of plasma-peptides for LC-MS/MS analysis should be limited for optimal performance of the LC-MS/MS.

Recently, a parallel effort to automate sample preparation on the OT-2 platform demonstrated equal reproducible performance for LC-MS/MS analysis but at lower throughput including overnight digestion at 37 °C and 42 min (30 SPD) LC gradients (25). In this platform, the OT-2 script generation is segmented into several subprocesses including a reduction/alkylation step, bead digestion, and finally Evotip loading, each step with individual OT-2 deck layouts and scripts. This provides a very flexible user implementation, which also includes preparation for BCA protein measurements, but does also requires manual intervention at several time points and sequential loading of scripts. In comparison, the OT-2 script obtained from the Evosep homepage is less flexible but without manual intervention after the beads has been added to the samples.

When analyzing clinical samples with LC-MS/MS, the biological naturally variability is high, and it is therefore important to minimize variation in the preanalytical sample handling and preparation. In the clinical cohort prepared on the OT-2 robot, we identify several known and some potentially novel biomarkers for clinical response to checkpoint inhibitors in malignant melanoma patients, and thereby demonstrate a great potential for biomarker discovery in clinical samples.

The addition of automated phosphopeptide enrichment using magnetic Ti-IMAC beads is integrated and only adds a few additional hours to the total run time. Although the total number of samples that can be processed is less than when preparing proteomes, the workflow enabled up to 48 proteomes and 48 phosphoproteomes in parallel allowing for streamlined cell signaling and systems biology investigations.

## Conclusion

Robust and reproducible automation of the sample preparation for bottom-up proteomics and phosphoproteomics is feasible and easily achieved on the Opentrons OT-2 robot. We demonstrate high performance and robustness in cancer cell lines and plasma of the automated system at high throughput. Integration of the digestion and sample loading on Evotips allows minimal hands-on time and a substantial decrease in the required sample input and reagents needed for digestion. Additionally, we also demonstrate the performance and potential for large-scale clinical biomarker and cellular signaling studies.

## Data Availability

The MS proteomics data have been deposited to the ProteomeXchange Consortium *via* the PRIDE partner repository with the dataset identifier PXD048325.

## Supplemental data

This article contains supplemental data.

## Conflict of interest

D. B. B.-J., N. B., M. H., and J. M. V.-N. are employees of Evosep Biosystems. The other authors declare no competing interest.

## Supplemental Data

Supplemental Table S1 HeLa protein list. List of protein groups identified uniprot id, gene symbol, protein name, median normalized label-free intensity, median log2 label-free intensity and dataset rank in 192 HeLa samples processed on the Opentrons OT-2 robot at and analysed on a Orbitrap Astral at a 100SPD (11.5 min) gradient. **Supplemental Table S2**. Plasma protein list. List of proteins groups identified with uniprot id, gene symbol, protein name, functional annotation (adapted from proteinatlas.org), FDA drug target designation and median label-free intensity at different evotip loads processed on the Opentrons OT-2 robot at and analysed on a Orbitrap Exploris at a 100 SPD (11.5 min) gradient. **Supplemental Table S3**. Protein list in clinical plasma samples. List of protein groups identified with uniprot id, gene symbol, protein name, median normalized label-free intensity in metastatic melanoma patient plasma samples before and after checkpoint inhibitor therapy divided in responders (R) and non-responders (NR) processed on the Opentrons OT-2 robot at and analysed on a Orbitrap Exploris at a 100 SPD (11.5 min) gradient.
